# Methodology of mixed load customized bus lines and adjustment based on time windows

**DOI:** 10.1371/journal.pone.0189763

**Published:** 2018-01-10

**Authors:** ZhiJie Li, Rui Song, Shiwei He, Mingkai Bi

**Affiliations:** 1 MOE Key Laboratory for Urban Transportation Complex Systems Theory and Technology, Beijing Jiaotong University, Beijing, China; 2 School of Traffic and Transportation, Beijing Jiaotong University, Beijing, China; Beihang University, CHINA

## Abstract

Custom bus routes need to be optimized to meet the needs of a customized bus for personalized trips of different passengers. This paper introduced a customized bus routing problem in which trips for each depot are given, and each bus stop has a fixed time window within which trips should be completed. Treating a trip as a virtual stop was the first consideration in solving the school bus routing problem (SBRP). Then, the mixed load custom bus routing model was established with a time window that satisfies its requirement and the result were solved by Cplex software. Finally, a simple network diagram with three depots, four pickup stops, and five delivery stops was structured to verify the correctness of the model, and based on the actual example, the result is that all the buses ran 124.42 kilometers, the sum of kilometers was 10.35 kilometers less than before. The paths and departure times of the different busses that were provided by the model were evaluated to meet the needs of the given conditions, thus providing valuable information for actual work.

## 1 Introduction

Because travel demand is relatively consistent, a public transportation service with a fixed route and timetable is suitable for daily life. However, with increasingly diversified economies, lifestyles, and time constraints, the methods and quality of travelling also tend to be diversified. Passengers will choose a suitable trip mode according to their travel time tolerance, economic capacity, comfort requirements, and even psychological preferences. However, traditional public transportation comes up short in terms of service innovation, service standards, and transport capacity. Many original ground public transportation services have suffered a massive loss of passengers; furthermore, regular bus services have lost passengers and long-distance travel has been at a disadvantage. Faced with increasingly diversified travel needs, a single homogenizing conventional public transport service will inevitably produce low vehicle turnover, unbalanced load rates, irrational choices regarding vehicle models, and other problems that result in high operating costs. The introduction of a customized bus business not only meets the needs of personalized passenger travel, but it also guides more individuals to give up private car travel, which can ease issues such as traffic jams.

Customized bus businesses are part of a novel public transit system that was first introduced and implemented in Qingdao in August 2013 [1]. However, the concept of vehicle sharing is not new, and the earliest car-sharing system started in Zurich in 1948 [2]. Kirby and Bhatt [3–4] discussed the subscription bus service, which is somewhat similar to the customized bus service, in the United States. McCall [5] analyzed the evolution and operations of a subscription commuter-bus-service system named COM-BUS that was successfully operated. Chang and Schonfeld [6] developed analytical optimization models to compare conventional and subscription bus systems that provided a feeder service to a single transportation terminal [7]. The State Council of the People's Republic of China released guidelines on the prior development of public transportation systems, especially about charter bus services, in 2012 [8]. Hu and Zhang [9] analyzed the background, definition, operation planning process, variables, and advantages of the customized bus service provided in Qingdao. After one month, a customized bus service was implemented in Beijing [10]. Xu et al. [11] discussed how the customized bus could enhance a city's PT system in China.

Ma et al. [12] proposed an efficient data-mining procedure that models the travel patterns of transit riders in Beijing while transit riders’ trip chains were identified based on the temporal and spatial characteristics of their smart card transaction data. The results of this procedure indicated that the proposed rough-set-based algorithm outperforms other commonly used data-mining algorithms with respect to accuracy and efficiency. Ma et al. [13] developed a series of data mining methods to identify the spatiotemporal commuting patterns of the Beijing public transit riders, and the findings provided useful insights for policymakers to shape a further balanced job-housing relationship by adjusting the monocentric urban structure of Beijing.

The customized bus operation-planning process commonly includes five basic activities that are usually performed in this sequence: network route design, timetable development, vehicle scheduling, crew scheduling, and real-time control [14–16]. Generally speaking, traditional methods for bus network route designs are based on historical passenger demand information, while the network route-design problem is typically formulated as a mathematical programming problem. Based on some predefined specific optimization parameters, techniques are employed to solve the optimization problem over the entire decision-making horizon [17]. Ma et al. [18], based on an analysis of domestic and international research, the current development of customized buses in China, and passenger travel data, established a model for the stop planning and timetables of customized buses. The improved immune genetic algorithm (IIGA) was used to solve the model as illustrated. The customized bus routing problem mentioned above was leveraged to solve the school bus routing problem (SBRP) in this paper [19–20] since it is just a more focused version of a vehicle routing problem (VRP). VRP problems [21] can generally be described as a vehicle starting from the depot, returning to the depot after providing delivery services to the customer, thus requiring that all of the customers are serviced, and that the customers must complete a delivery under the capacity constraints of the vehicle in order to achieve the purpose of the minimum total distance. The SBRP is based on that of the VRP, and the demand point can be divided into student and school stops, a certain time window, vehicle capacity, transport distance, etc. in order to determine which students should be serviced by the bus and the number of the school buses needed. This organization can achieve the purpose of minimum total cost.

Around the world, there are numerous studies on the school bus routing problem. In order to solve the school bus routing problem for a single school and a single vehicle model, Bennett et al. [22] took the shortest total distance travelled by the bus as the end goal. They started by generating the path of the school bus, and through the improvement of the C-W savings algorithm, solved the problem with a size of 256 depots. Angel et al. [23] put forward the concept of "clustering structure after the first path" for the school bus routing problem without a mixed load and took the shortest total distance as the goal. The stops were first clustered, then according to the TSP(Travelling Salesman Problem), merged again to construct the vehicle routing. Park et al. [24] solved the school bus routing problem with a mixed loading mode of students, thus proposing an improved algorithm that proved to be a benchmark in this research type. Kim et al. [25] studied the school bus routing problem with time windows and found that each different school can be serviced by the same school bus many times, while the heuristic algorithm was compared based on the benchmark instance. Euchi et al. [26] took into consideration when the school bus arrived at the students’ stop, then took the minimum distance of the vehicle as a target, and studied the city school bus routing problem in Tunisian City with the variable neighborhood search algorithm and ant colony optimization. Fu [27] constructed a multi-objective bus route model of a multi-type bus and proposed a heuristic algorithm of the farthest person serviced first, similar to the method when the stop farthest from the school must be assigned before assigning the rest of the stops, to adjust to meeting the demand. Ding [28] took the multi-center SBRP and converted it into a single-center SBRP by adding a virtual depot and obtained the results using the colony algorithm.

Overall, the research done on the school bus routing problem around the world mostly belongs to a single SBRP [29]. A single school SBRP can be solved by constructing the model with a time window and vehicle capacity constraint [30], but there has been relatively less research on the mixed load SBRP. A custom bus routing problem is similar to the SBRP since each bus must pick up passengers from their homes or bus stop and transfer them to their company or a different bus stop, while satisfying various constraints, such as bus capacity, passengers’ maximum allowable riding time in a bus, and time window constraints of the companies. If buses are dedicated to a specific company, the problem becomes a vehicle routing problem (VRP). However, in general customized bus routes can serve passengers from different companies so a bus can visit several companies. It is assumed that “mixed load is allowed” if passengers from different companies can be put on the same bus at the same time, but the mixed load model must be considered more deeply than this. The passengers of different pickup locations and different destinations should be mixed by arranging different busses with a customized bus, and the passengers must be sent to the destination in the specified time window of the delivery stop.

Because of the late development of the customized bus, there is little research on customized busses; therefore, in this paper the customized bus routing problem needs further research on the basis of predecessors' research regarding the SBRP. Then, the mixed load customized bus routing model will be established with a time window that satisfies its requirement, and the result will be solved by Cplex software.

## 2 Route optimization model of mixed load customized bus with time window

### 2.1 Problem description

The customized bus routing problem of the paper can be expressed as a bus company having a certain number of depots, while each depot has a number of customized business buses. There are many stops in addition to depots. Stops are divided into pickup stops and delivery stops according to the behavior of passengers, where pickup stops and delivery stops only allow passengers to get on and get off, respectively. When opening the route, the bus company is required to investigate and count the needs of passengers who are travelling, and then design routes according to travelling needs and passenger flow. In each decision cycle a bus starts from a depot, selects a time window from the pickup stop, delivers passengers to scheduled and time-limited delivery stops, and finally comes back to the nearest distribution center. During the decision cycle, the requirements of each stop can be met within a defined time window.

As for addressing the problem of business bus route optimization, it can be regarded as a vehicle routing problem with a time window, multiple centers, and mixed loads. Transferring is not included since it aims to achieve "point-to-point" direct transportation. Reasonable allocation of vehicles in the depots was selected and suitable transportation routes were arranged to meet the passenger's personalized travel demand and time window restrictions in order to reduce operation and time costs.

### 2.2 Modelling assumption

To simplify the problem, some hypothetical assumptions are made:

(1) The number of passengers at the pickup and delivery stops is known; (2) The distance from the depot to each stop and one stop to another stop is known; (3) The model of the buses is the same, i.e., the same passenger capacity; (4) Each bus only runs one route and starts from the depot and ends at the same place; (5) A single peak, namely morning peak or evening peak, without considering a two-way peak at the same time; (6) The distance between the nodes is symmetrical; (7) The depot is owned by the same company and can be freely used; (8) Each vehicle is driven under ideal road condition, regardless of road resistance; (9) “One seat one passenger” mode is applied in the bus, therefor no standing passengers; (10) Passengers can only get on at a pickup stop and get off at a delivery stop; (11) No passengers get on or off at the depot; and (12) The time window of the vehicle to each stop is equal.

### 2.3 Relevant parameter variable definition

Suppose that the number of bus stops is *v*. It is assumed that the passenger is picked up from node *i*. Companies are duplicated in order to formulate our problem as a pickup and delivery like model. The model includes the following parameters and decision variables: let *D*_*e*_ be a set of pickup location (bus stops) and *D*_*s*_ be a set of delivery locations or companies, and *D* ∈ *D*_*e*_ ∪ *D*_*s*_. There is also a set *k* for company buses with the same capacity *D*. Each bus *k* starts at a predetermined starting location *m* and returns at an ending location *m*, and set *V* is a union of sets *M* and *D* (*V* = *M*∪*D*). Rectilinear distance from node *i* to *j* (*i*,*j* ∈ *V*) is *d*_*ij*_. Each pickup and delivery stop has an earliest *E*_*i*_ and latest possible arrival time *L*_*i*_, respectively, and the passengers should arrive at the pickup and delivery stops between *E*_*i*_ and *L*_*i*_. The student’s maximum riding time in a bus is given by *R*. Note that the maximum riding time usually depends on the pickup and delivery location. A bus can visit some stops, but each stop should not be visited more than once by the same bus.

In the mathematical model, the definition of symbols and implications used in the model are shown in S1 Table:

### 2.4 Route optimization of customized business bus with soft window

Using these parameters and variables, the mixed load customized bus route problem is formulated with soft window as a mixed integer programming (MIP) model. Note that the mixed customized bus route problem can be formulated as a model similar to the pickup and delivery problem with time window (PDPTW). The PDPTW aims to find a set of optimal routes to serve the transportation requests of the customers [31]. Each request is picked up at the origin node and delivered to the destination node of the request. The bus must visit the pickup nodes and the corresponding delivery nodes. From the perspective of business managers, they expect to stratify personalized traveling with a customized business bus; therefore, to minimize the operation cost an MIP function for our problem is shown as Eq (1):

$$Min\;f=c_{1}\sum_{k\in K}\sum_{i\in V}\sum_{j\in V}d_{ij}y_{ij}^{k}$$ (1)

$$\sum_{k\in K}x_{i}^{k}\geq 1,\;i\in D$$ (2)

$$\sum_{i\in M}y_{iu}^{k}-\sum_{j\in M}y_{uj}^{k}=0,\;\forall u\in D,\;k\in K$$ (3)

$$\sum_{k\in K}H_{i}^{k}=n_{i},\;i\in D$$ (4)

$$0\leq H_{i}^{k}\leq Q,\;i\in D,k\in K$$ (5)

$$H_{i}^{k}\leq n_{i}\sum_{j\in D}y_{ij}^{k},\;i\in D_{e},j\neq i,k\in K$$ (6)

$$H_{i}^{k}\geq \sum_{j\in D}y_{ij}^{k},\;i\in D_{e},j\neq i,k\in K$$ (7)

$$\sum_{k\in K}\sum_{j\in D_{e}}y_{ij}^{k}\leq 1,\;i\in M$$ (8)

$$T_{i}^{k}+y_{ij}^{k}\left(st_{i}+\frac{d_{ij}}{v_{s}}\right)\leq T_{j}^{k},\;i\in D_{e},j\in D_{s},k\in K$$ (9)

$$y_{ij}^{k}\leq x_{j}^{k},\;i\in D,j\in D,j\neq i,k\in K$$ (10)

$$x_{i}^{k}\leq H_{i}^{k},\;i\in D,k\in K$$ (11)

$$\sum_{i\in D}x_{i}^{k}\leq f,\;k\in K$$ (12)

$$\sum_{i\in D_{e}}p_{ij}y_{ij}^{k}\leq n_{j},\;j\in D_{s},k\in K$$ (13)

$$\sum_{j\in D_{s}}p_{ij}y_{ij}^{k}\leq n_{i},\;i\in D_{e},k\in K$$ (14)

$$\sum_{i\in D_{e}}H_{i}^{k}<=Q,\;k\in K$$ (15)

$$\sum_{j\in D_{s}}H_{j}^{k}<=Q,\;k\in K$$ (16)

$$0<T_{j}^{k}-T_{i}^{k}\leq R,\;i\in D_{e},j\in D_{s},k\in K$$ (17)

$$E_{i}\leq T_{i}^{k}\leq L_{i},\;i\in D,k\in K$$ (18)

$$T_{d(k)}^{k}+\frac{d_{d(k)j}}{v_{s}}\leq T_{j}^{k},\;j\in D_{e},k\in K$$ (19)

$$x_{i}^{k}\in \left\{0,1\right\},\;i\in D,k\in K$$ (20)

$$y_{ij}^{k}\in \left\{0,1\right\},\;i\in V,j\in V,k\in K$$ (21)

$$T_{i}^{k}\geq 0,\;i\in D,k\in K$$ (22)

$$H_{i}^{k}\geq 0,\;i\in D,k\in K$$ (23)

Constraints (2) ensure that each stop can be serviced by several buses within each time window, and that each stop can be serviced. Constraints (3) ensure that each bus must start from the bus depot and return to the depot after transporting passengers. Constraints (4) indicate that the number of passengers on or off different buses that are passing through each stop is equal to the number of passengers at the stop. Constraints (5) indicate that the number of passengers is limited by different busses that are passing through each stop. Constraints (6) and (7) ensure that only when the bus passes the pickup stop, the pickup stop can be served by the bus, and the result is $0<H_{i}^{k}\leq n_{i}^{k}$, otherwise $H_{i}^{k}=0$. Constraints (8) indicate that the Kth bus can leave the depot only one time. Constraints (9) are the order of bus visiting constraints, ensuring that the bus must first visit a pickup stop before it can visit a corresponding delivery stop. Constraints (10) and (11) show the relationship between the two variables. Constraints (12) ensures that the number of nodes served by a bus is less than or equal to the total number of nodes. Constraints (13) and (14) show the relationship between the stop and the specified stop. Constraints (15) and (16) indicate that the number of passengers on the bus should not exceed its capacity after each bus has passed a pickup or delivery stop. Constraints (17) are the time constraint from a pickup stop to a delivery stop. Constraints (18) ensure that the vehicle must arrive at the stop within each defined time window. Constraints (19) are the relationship between the departure time and arrival time. Constraints (20) and (21) are 0–1 constraints meaning that the limit value can only be 0 or 1. Constraints (22) are a nonnegative constraint as the limit value can only take a nonnegative number. Constraints (23) are a nonnegative integer constraint; the limit value can only take 0 or a positive integer.

## 3 Case study

### 3.1 Case verification

In this paper, a simple network diagram is constructed with three depots, nine pickup stops, and six delivery stops for a total of eighteen nodes, as shown in S1 Fig. The three depots are depot A, depot B, and depot C. For convenience, the nine pickup stops are set as 1, 2, 3, 4, 5, 6, 7, 8, and 9, while the six delivery stops are 10, 11, 12, 13, 14, and 15. The passengers at the nine bus stops have different travel requirements for the six delivery stops, as shown with the blue line in S1 Fig., indicating that the first bus departs from depot A and traverses to pickup stop 1, 2 and then 6. Within the first time window specified by the stop, passengers at stop 1, 2, and 6 are likely to get on and off at stops 10, 11, and 12. After delivering all of the passengers, the bus will leave for depot C. Another route with different colors is also shown with a similar situation.

The parameters are assigned according to the model established above. It is assumed that there are three depots and six buses. One to four buses belong to depot A, and the departure times are 7:00, 7:02, 7:03, and 7:05 a.m. Five to six buses belong to depot B, and departure times are 6:54 and 6:57 a.m. There is no departure bus in depot C. The unit distance cost *c*_1_ is 10 yuan/km, and the average speed, *V*_*s*_, of the bus is 60 km/hour. The number of passengers from stop *i* to specified stop *j* is shown in S2 Table.

The distance between nodes, *d*_*ij*_, is shown in S3 Table. The rated passenger capacity of bus *Q*_*k*_ is 40 people. The number of passengers at stop *i* is shown in S4 Table. The maximum riding time is 3 hours, while the waiting time of the bus at stop *i* is 30 seconds. The earliest and latest service time of stop *i* is shown in S5 Table.

Ilog Cplex software can be used to solve the given MIP model, and it has been used to solve various other types of transportation problems. Solving software Ilog Cplex was developed by IBM and has great advantages in solving mixed integer programming, while producing accurate and efficient solutions to problems. With regard to the specific calculation process and parameter determination, the parameters of Cplex are mainly explained in the document [32]. The model is a mixed integer linear optimization model, which can be solved by an exact solution or a heuristic algorithm. Ilog Cplex software’s embedded algorithm can be set according to the needs of solving self-configurations, such as single optimization procedures, limit optimization programs, and mixed integer optimization procedures. In this paper, the route optimization model of mixed load customized bus with time window is MIP model, the branch and bound algorithm was uesd to solve this problem, that is how to slove LP problem, the key to solving the MIP problem was to decompose the problem into many subproblems, even if the small MIP problem needed a lot of computation. After getting the optimal solution by the general linear programming method, decision variables should be splited into two integers, then the optimal solution can be got after the upper bound (upper bound) or lower bound (lower bound) of the objective function value was obtained. In addition, in order to better deal with the analysis and calculation results, this paper uses the C# program to call the Cplex algorithm solver to solve the model.

IBM Cplex 12.5 was used and the computational results were completed using the hardware configuration of an i5-2410 MCPU and 4G of RAM. The results of the model were calculated in only approximately 3 seconds, and the results are shown in S6 Table. The objective function obtained an optimal solution of 2,400 yuan.

The results of the operation show the different bus routes that can be seen in S7 Table. The first through the sixth busses depart from their respective depots according to the given departure time. Then, the route of each bus and the number of passengers getting on or off the different buses that are passing through each stop can be obtained with the distance between all of the depots and stops. The number of passengers who get on or off at the stop, and the specified time window of the stop can also be seen in S7 Table. The route of the first bus was A→2→6→10→13→C, the bus ran 37 kilometers, and held 26 people in total. There were 7 and 19 people who got on at the 2^nd^ and 6^th^ pickup stops, respectively, and 15 and 11 people who got off at the 10^th^ and 13^th^ delivery stops, respectively. The route of the second bus was A→2→9→11→13→14→C, the bus ran 42 kilometers, and held 40 people in total. There were 10 and 30 people who got on at the 2^nd^ and 8^th^ pickup stops, respectively, and 17, 4, and 19 people who got off at the 11^th^, 13^th^ and 14^th^ delivery stops, respectively. The route of the third bus was A→4→11→12→B, the bus ran 35 kilometers, and held 40 people in total. There were 40 people who got on the 4^th^ pickup stop, and 11 and 29 people who got off at the 11^th^ and 12^th^ delivery stops, respectively. The route of the 4th bus was A→2→9→14→C, the bus ran 37 kilometers, and held 40 people in total. There were 15 and 25 people who got on at the 2^nd^ and 9^th^ pickup stops, respectively, and 40 people who got off the 14^th^ delivery stop. The route of the 5^th^ bus was B→3→7→9→13→C, the bus ran 42 kilometers, and held 40 people in total. There were 35, 4, and 1 people who got on at the 3^rd^, 7^th^, and 9^th^ pickup stops, respectively, and 40 people who got off at the 13^th^ delivery stop. The route of the 6^th^ bus was B→1→2→5→11→15→C, the bus ran 47 kilometers, and held 40 people in total. There were 14, 1, and 25 people who got on at the 1^st^, 2^nd^ and 5^th^ pickup stops, respectively, and 9 and 31 people who got off the 10^th^ and 15^th^ delivery stops, respectively.

Since the 5th and 6th busses left from depot B, the distance from depot B to the pickup stop was relatively too long. Therefore, when the 5th and 6th busses started at 6:54 and 6:57, the arrival time at the pickup stop was later; then, the remaining passengers at the stop were transported and finally went to the nearest depot.

### 3.2 Actual case

This paper takes Guan Zhuang residential district to Wang Jing trading area as an example, Guan Zhuang residential district includes East of Chaoyang Mong Kok District, Ocean Side, Run Yuan of Ocean Side, Hua Yu Yuan of Ocean Side, East of Ta Ying Street, Small Temple and West of Shuang Qiao, total 7 pickup stop. Wang Jing trading area includes Guangze Road, South of Da Yu Zi Intersection, Wang Ye Fen, South of Guang Shun Street, East of Fu Tong Street, Guo Feng Beijing, Rong Ke Gan Lan City, West of Bei Xiao He, Hong Tai East Stree, Bei Xiao He and East of Wang Jing Bei Road, total 11 delivery stop, the **name of up and down station** is shown in S8 Table. The customized bus starts directly from the East of Chaoyang Mong Kok District and Ocean Side.

There are 3 buses in the Ocean Side stop and 2 buses in the East of Chaoyang Mong Kok District stop, the departure times in the Ocean Side stop and East of Chaoyang Mong Kok District stop are 7:20 a.m. and 7:40 a.m., and the rated passenger capacity of bus is 49 people.

The unit distance cost *c*_1_ is 10 yuan/km, and the average speed, *V*_*s*_, of the bus is 40 km/hour, the maximum riding time is 1.5 hours, while the waiting time of the bus at stop *i* is 30 seconds. The distance between nodes, *d*_*ij*_, is shown in S9 Table.

The number of passengers from stop *i* to specified stop *j* is shown in S10 Table. The number of passengers getting on or off in the time window is shown in S11 Table.The earliest and latest service time of stop *i* is shown in S12 Table. According to the serial number of S8 Table, the earliest service time of stop is 7:40, 7:20, 7:42, 7:44, 7:46, 7:48, 7:44, 8:25, 7:52, 7:51, 7:52, 7:54, 7:55, 7:58, 8:04, 8:05, 8:10, 8:15 respectively, the latest service time of stop is 7:42, 7:22, 7:52, 7:54, 7:56, 7:58, 7:54, 8:50, 7:58, 7:59, 8:00, 8:03, 8:05, 8:10, 8:15, 8:25, 8:30, 8:45.

The results of the operation show the different bus routes that can be seen in S12 Table. The first bus ran 32.72 kilometers, and held 49 people in total. The second bus ran 23.44 kilometers, and held 49 people in total. The third bus ran 23.47 kilometers, and held 49 people in total. The fourth bus ran 23.78 kilometers, and held 49 people in total. The fifth bus ran 32.72 kilometers, and held 49 people in total. All the buses ran 124.42 kilometers, the sum of kilometers was 10.35 kilometers less than before. Moreover, the planned lines can be allocated according to the needs of the stop, instead of the exorbitant bus line repetition rate.

## 4 Conclusions

Customized bus lines were optimized to meet the needs of personalized travel for different passengers. On the basis of the traditional vehicle routing model, customized bus routing was considered as a path selection problem under a time window constraint The mode of the pickup and delivery location separation was also considered, while the mixed load customized bus routing model had to be established with a time window that satisfied its requirement. Then, the problem model was solved by Cplex software.

Taking into consideration the virtual network of two depots and eight domain points, the paths of different busses and different departure times were obtained that met the needs of the given conditions. The results had a certain value and a reference function to make actual route planning of a customized bus simple and less time consuming.

## Supporting information

S1 Fig Simple Network Diagram. (TIF)

S1 Table Model Symbol Definition. (DOCX)

S2 Table Number of Passengers From Stop *i* to Specified Stop *j*. (DOCX)

S3 Table Distance Between Nodes. (DOCX)

S4 Table Number of Passengers Getting on or off in the Time Window. (DOCX)

S5 Table Earliest and latest Service Times of Stop *i*. (DOCX)

S6 Table The Optimal Solution of the Model. (DOCX)

S7 Table The Optimal Result of the Model. (DOCX)

S8 Table Name of Up and Down Station. (DOCX)

S9 Table Distance Between Nodes. (DOCX)

S10 Table Number of Passengers From Stop *i* to Specified Stop *j*. (DOCX)

S11 Table Number of Passengers Getting on or off in the Time Window. (DOCX)

S12 Table The Optimal Result of the Model. (DOCX)
