# Supplementary figures and images for: Methodology of mixed load customized bus lines and adjustment based on time windows
Source: PLoS One. 2018 Jan 10;13(1):e0189763. doi: 10.1371/journal.pone.0189763 (PMC5761835; doi:10.1371/journal.pone.0189763)

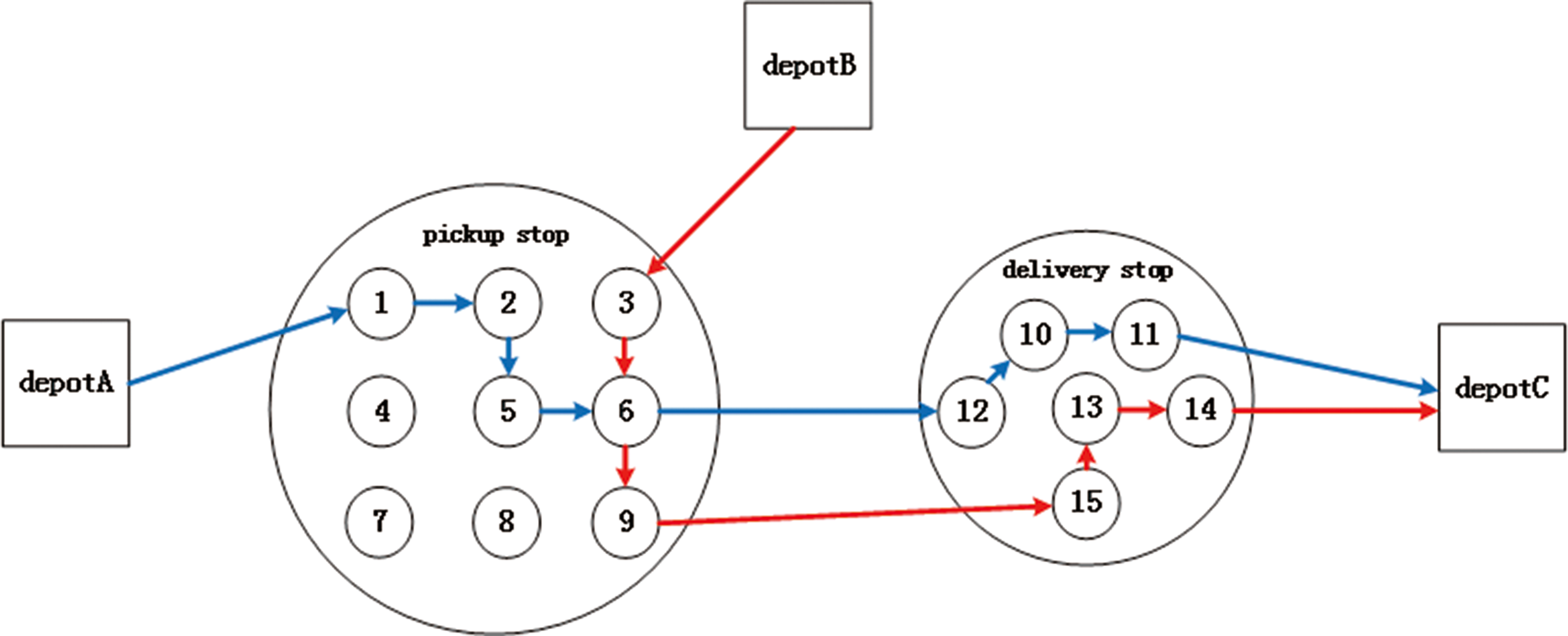

Supplement: S1 Fig — (TIF) [file pone.0189763.s001.tif]
