# Supplementary material for: Methodology of mixed load customized bus lines and adjustment based on time windows
Source: PLoS One. 2018 Jan 10;13(1):e0189763. doi: 10.1371/journal.pone.0189763 (PMC5761835; doi:10.1371/journal.pone.0189763)
Supplement: S1 Table — (DOCX) [file pone.0189763.s002.docx]

**S1 Table. Model Symbol Definition.**

| **Assemble** | |
| --- | --- |
| $M$ | bus depot assemble,$m\in M$ |
| $K$ | bus number assemble$k\in K$ |
| $D_{e}$ | passenger at pickup stop assemble |
| $D_{s}$ | passenger at delivery stop assemble |
| $D$ | all stops assemble,$D\in D_{e}\cup D_{s}$ |
| $V$ | all nodes assemble,$V\in D\cup M$ |
| **Parameter** | |
| $c_{1}$ | unit distance delivery cost |
| $d_{ij}$ | distance between stop $i$ and $j$, $i,j\in V$ |
| $Q_{k}$ | specified passenger capacity of bus $k$ |
| $n_{i}$ | number of passenger picked up or delivered of stop $i$ |
| $p_{ij}$ | number of passenger from stop $i$ to specified stop $j$ |
| $R$ | maximum riding time of passenger |
| $v_{s}$ | average running speed from $i$ to $j$ |
| $st_{i}$ | residence time at stop $i$ |
| $f$ | total number of node |
| $d\left(k\right)$ | depot of the Kth bus, $k\in K$ |
| $T_{0}^{k}$ | departure time of Kth bus from depot |
| $E_{i}$ | earliest service time of stop $i$ |
| $L_{i}$ | latest service time of stop $i$ |
| **Decision Variables** | |
| $x_{i}^{k}$ | 0-1 variable, if stop $i$ is selected by the kth bus, the value is 1, otherwise is 0. |
| $y_{ij}^{k}$ | 0-1 variable, if the Kth bus drives from $i$ to $j$, the value is 1, otherwise is 0. |
| $T_{i}^{k}$ | arrival time at stop $i$ |
| $H_{i}^{k}$ | number of passengers on or off the stop after the bus arrives at stop $i$ |
