# Supplementary material for: Methodology of mixed load customized bus lines and adjustment based on time windows
Source: PLoS One. 2018 Jan 10;13(1):e0189763. doi: 10.1371/journal.pone.0189763 (PMC5761835; doi:10.1371/journal.pone.0189763)
Supplement: S3 Table — (DOCX) [file pone.0189763.s004.docx]

**S3 Table. Distance Between Nodes.**

| **stop** | **1** | **2** | **3** | **4** | **5** | **6** | **7** | **8** | **9** | **10** | **11** | **12** | **13** | **14** | **15** | **depotA** | **depotB** | **depotC** |
| --- | --- | --- | --- | --- | --- | --- | --- | --- | --- | --- | --- | --- | --- | --- | --- | --- | --- | --- |
| **1** | 0 | 2 | 3 | 2 | 3 | 5 | 5 | 6 | 7 | 15 | 17 | 14 | 16 | 19 | 15 | 8 | 10 | 24 |
| **2** | 2 | 0 | 2 | 2 | 4 | 4 | 6 | 7 | 6 | 14 | 16 | 13 | 15 | 18 | 14 | 9 | 9 | 23 |
| **3** | 3 | 2 | 0 | 3 | 4 | 4 | 7 | 5 | 5 | 13 | 15 | 12 | 14 | 17 | 13 | 10 | 8 | 22 |
| **4** | 2 | 2 | 3 | 0 | 3 | 4 | 4 | 5 | 6 | 14 | 16 | 13 | 15 | 18 | 14 | 7 | 11 | 24 |
| **5** | 3 | 4 | 4 | 3 | 0 | 4 | 4 | 5 | 6 | 13 | 15 | 12 | 14 | 17 | 13 | 8 | 10 | 23 |
| **6** | 5 | 4 | 4 | 4 | 4 | 0 | 6 | 5 | 5 | 11 | 13 | 10 | 12 | 15 | 11 | 9 | 9 | 21 |
| **7** | 5 | 6 | 7 | 4 | 4 | 6 | 0 | 5 | 6 | 12 | 14 | 11 | 13 | 16 | 12 | 8 | 13 | 24 |
| **8** | 6 | 7 | 5 | 5 | 5 | 5 | 5 | 0 | 4 | 10 | 12 | 9 | 11 | 14 | 10 | 9 | 11 | 22 |
| **9** | 7 | 6 | 5 | 6 | 6 | 5 | 6 | 4 | 0 | 9 | 11 | 9 | 11 | 13 | 9 | 10 | 10 | 21 |
| **10** | 15 | 14 | 13 | 14 | 13 | 11 | 12 | 10 | 9 | 0 | 2 | 2 | 3 | 4 | 5 | 14 | 8 | 10 |
| **11** | 17 | 16 | 15 | 16 | 15 | 13 | 14 | 12 | 11 | 2 | 0 | 3 | 3 | 4 | 6 | 15 | 9 | 8 |
| **12** | 14 | 13 | 12 | 13 | 12 | 10 | 11 | 9 | 9 | 2 | 3 | 0 | 3 | 5 | 4 | 12 | 9 | 11 |
| **13** | 16 | 15 | 14 | 15 | 14 | 12 | 13 | 11 | 11 | 3 | 3 | 3 | 0 | 4 | 3 | 14 | 10 | 10 |
| **14** | 19 | 18 | 17 | 18 | 17 | 15 | 16 | 14 | 13 | 4 | 4 | 5 | 4 | 0 | 4 | 16 | 11 | 9 |
| **15** | 15 | 14 | 13 | 14 | 13 | 11 | 12 | 10 | 9 | 5 | 6 | 4 | 3 | 4 | 0 | 14 | 11 | 10 |
| **depotA** | 8 | 9 | 10 | 7 | 8 | 9 | 8 | 9 | 10 | 14 | 15 | 12 | 14 | 16 | 14 | 0 | 16 | 29 |
| **depotB** | 10 | 9 | 8 | 11 | 10 | 9 | 13 | 11 | 10 | 8 | 9 | 9 | 10 | 11 | 11 | 16 | 0 | 16 |
| **depotC** | 24 | 23 | 22 | 24 | 23 | 21 | 24 | 22 | 21 | 10 | 8 | 11 | 10 | 9 | 10 | 29 | 16 | 0 |
