# Supplementary material for: Methodology of mixed load customized bus lines and adjustment based on time windows
Source: PLoS One. 2018 Jan 10;13(1):e0189763. doi: 10.1371/journal.pone.0189763 (PMC5761835; doi:10.1371/journal.pone.0189763)
Supplement: S4 Table — (DOCX) [file pone.0189763.s005.docx]

**S4 Table. Number of Passengers Getting on or off in the Time Window.**

| **Stop** | **1** | **2** | **3** | **4** | **5** | **6** | **7** | **8** | **9** | **10** | **11** | **12** | **13** | **14** | **15** |
| --- | --- | --- | --- | --- | --- | --- | --- | --- | --- | --- | --- | --- | --- | --- | --- |
|  | 14 | 33 | 35 | 40 | 25 | 19 | 4 | 30 | 26 | 15 | 37 | 29 | 55 | 59 | 31 |
