# Supplementary material for: Methodology of mixed load customized bus lines and adjustment based on time windows
Source: PLoS One. 2018 Jan 10;13(1):e0189763. doi: 10.1371/journal.pone.0189763 (PMC5761835; doi:10.1371/journal.pone.0189763)
Supplement: S5 Table — (DOCX) [file pone.0189763.s006.docx]

**S5 Table. Earliest and Latest Service Times of Stop $i$.**

| **Stop**  **Time** | **1** | **2** | **3** | **4** | **5** | **6** | **7** | **8** | **9** | **10** | **11** | **12** | **13** | **14** | **15** |
| --- | --- | --- | --- | --- | --- | --- | --- | --- | --- | --- | --- | --- | --- | --- | --- |
| **The earlist service time** | **7:04** | **7:03** | **7:02** | **7:03** | **7:04** | **7:03** | **7:07** | **7:05** | **7:04** | **7:25** | **7:26** | **7:25** | **7:27** | **7:28** | **7:26** |
| **The latest service time** | **7:12** | **7:14** | **7:14** | **7:12** | **7:13** | **7:13** | **7:12** | **7:13** | **7:15** | **7:35** | **7:36** | **7:35** | **7:37** | **7:38** | **7:36** |
