# Supplementary material for: Methodology of mixed load customized bus lines and adjustment based on time windows
Source: PLoS One. 2018 Jan 10;13(1):e0189763. doi: 10.1371/journal.pone.0189763 (PMC5761835; doi:10.1371/journal.pone.0189763)
Supplement: S6 Table — (DOCX) [file pone.0189763.s007.docx]

**S6 Table. The Optimal Solution of the Model.**

|  | **1st bus** | | **2nd bus** | | **3rd bus** | | **4th bus** | | **5th bus** | | **6th bus** | |
| --- | --- | --- | --- | --- | --- | --- | --- | --- | --- | --- | --- | --- |
| **stop** | **Delivery number** | **arrival time** | **delivery**  **number** | **arrival time** | **delivery**  **number** | **arrival time** | **delivery**  **number** | **arrival time** | **delivery**  **number** | **arrival time** | **delivery**  **number** | **arrival time** |
| **1** | 0 | / | 0 | / | 0 | / | 0 | / | 0 | / | 14 | 7.07 |
| **2** | 7 | 7.09 | 10 | 7.11 | 0 | / | 15 | 7.14 | 0 | / | 1 | 7.06 |
| **3** | 0 | / | 0 | / | 0 | / | 0 | / | 35 | 7.02 | 0 | / |
| **4** | 0 | / | 0 | / | 40 | 7.1 | 0 | / | 0 | / | 0 | / |
| **5** | 0 | / | 0 | / | 0 | / | 0 | / | 0 | / | 25 | 7.07 |
| **6** | 19 | 7.09 | 0 | / | 0 | / | 0 | / | 0 | / | 0 | / |
| **7** | 0 | / | 0 | / | 0 | / | 0 | / | 4 | 7.07 | 0 | / |
| **8** | 0 | / | 30 | 7.1 | 0 | / | 0 | / | 0 | / | 0 | / |
| **9** | 0 | / | 0 | / | 0 | / | 25 | 7.146 | 1 | 7.04 | 0 | / |
| **10** | 15 | 7.25 | 0 | / | 0 | / | 0 | / | 0 | / | 0 | / |
| **11** | 0 | / | 17 | 7.36 | 11 | 7.36 | 0 | / | 0 | / | 9 | / |
| **12** | 0 | / | 0 | / | 29 | 7.35 | 0 | / | 0 | / | 0 | / |
| **13** | 11 | 7.27 | 4 | 7.27 | 0 | / | 0 | / | 40 | 7.27 | 0 | / |
| **14** | 0 | / | 19 | 7.38 | 0 | / | 40 | 7.38 | 0 | / | 0 | / |
| **15** | 0 | / | 0 | / | 0 | / | 0 | / | 0 | / | 31 | 7.26 |

Note: S6 Table "/" indicates that the vehicle has not passed the point, there is no time
