# Supplementary material for: Methodology of mixed load customized bus lines and adjustment based on time windows
Source: PLoS One. 2018 Jan 10;13(1):e0189763. doi: 10.1371/journal.pone.0189763 (PMC5761835; doi:10.1371/journal.pone.0189763)
Supplement: S7 Table — (DOCX) [file pone.0189763.s008.docx]

**S7 Table. The Optimal Result of the Model.**

| **stop** | **Visiting order of stop** | **Cumulative operating distance (km)** | **Cumulative capacity (people)** |
| --- | --- | --- | --- |
| **1st bus** | A→2→6→10→13→C | 37 | 26 |
| **2nd bus** | A→2→9→11→13→14→C | 42 | 40 |
| **3rd bus** | A→4→11→12→B | 35 | 40 |
| **4th bus** | A→2→9→14→C | 37 | 40 |
| **5th bus** | B→3→7→9→13→C | 42 | 40 |
| **6th bus** | B→1→2→5→11→15→C | 47 | 40 |
