# Supplementary material for: Methodology of mixed load customized bus lines and adjustment based on time windows
Source: PLoS One. 2018 Jan 10;13(1):e0189763. doi: 10.1371/journal.pone.0189763 (PMC5761835; doi:10.1371/journal.pone.0189763)
Supplement: S8 Table — (DOCX) [file pone.0189763.s009.docx]

**S8 Table. Name of Up and Down Station.**

| date | Serial number | Pickup Stop |  | Delivery Stop |
| --- | --- | --- | --- | --- |
| 2016.9.9 | 1 | East of Chaoyang Mong Kok District | 8 | Guangze Road |
|  | 2 | Ocean Side | 9 | South of Da Yu Zi Intersection |
|  | 3 | Run Yuan of Ocean Side | 10 | Wang Ye Fen |
|  | 4 | Hua Yu Yuan of Ocean Side | 11 | South of Guang Shun Street |
|  | 5 | East of Ta Ying Street | 12 | East of Fu Tong Street |
|  | 6 | Small Temple | 13 | Guo Feng Beijing |
|  | 7 | West of Shuang Qiao | 14 | Rong Ke Gan Lan City |
|  |  |  | 15 | West of Bei Xiao He |
|  |  |  | 16 | Hong Tai East Street |
|  |  |  | 17 | Bei Xiao He |
|  |  |  | 18 | East of Wang Jing Bei Road |
