# Supplementary material for: Methodology of mixed load customized bus lines and adjustment based on time windows
Source: PLoS One. 2018 Jan 10;13(1):e0189763. doi: 10.1371/journal.pone.0189763 (PMC5761835; doi:10.1371/journal.pone.0189763)
Supplement: S9 Table — (DOCX) [file pone.0189763.s010.docx]

**S9 Table. Distance Between Nodes.**

| **stop** | **1** | **2** | **3** | **4** | **5** | **6** | **7** | **8** | **9** | **10** | **11** | **12** | **13** | **14** | **15** | **16** | **17** | **18** |
| --- | --- | --- | --- | --- | --- | --- | --- | --- | --- | --- | --- | --- | --- | --- | --- | --- | --- | --- |
| **1** | 0 | 2.1 | 18 | 2.1 | 2.6 | 2.9 | 2.5 | 1.7 | 15.4 | 15 | 15.8 | 16.5 | 16.9 | 16.9 | 18.5 | 16.4 | 19.1 | 18.7 |
| **2** | 2.1 | 0 | 19.4 | 0.36 | 0.58 | 0.9 | 0.44 | 2.3 | 16.9 | 16.3 | 17.2 | 17.8 | 18.3 | 18.3 | 19.7 | 17.7 | 20.5 | 20.1 |
| **3** | 18 | 19.4 | 0 | 19.3 | 19.9 | 19.8 | 19.4 | 17.2 | 2.6 | 3.0 | 2.5 | 1.9 | 1.5 | 1.5 | 0.97 | 1.9 | 1.9 | 1.0 |
| **4** | 2.1 | 0.36 | 19.3 | 0 | 0.6 | 0.92 | 0.47 | 2.3 | 16.9 | 16.3 | 17.2 | 17.8 | 18.3 | 18.3 | 19.7 | 17.7 | 20.5 | 20.1 |
| **5** | 2.6 | 0.58 | 19.9 | 0.6 | 0 | 0.33 | 0.57 | 2.8 | 17.4 | 16.9 | 17.7 | 18.3 | 18.7 | 18.7 | 19.9 | 18.2 | 21 | 20.2 |
| **6** | 2.9 | 0.9 | 19.8 | 0.92 | 0.33 | 0 | 0.47 | 3.2 | 17.6 | 17.1 | 18 | 18.6 | 18.7 | 18.7 | 19.8 | 18.1 | 21.3 | 20.1 |
| **7** | 2.5 | 0.44 | 19.4 | 0.47 | 0.57 | 0.47 | 0 | 2.7 | 17.2 | 16.7 | 17.5 | 18.1 | 18.2 | 18.2 | 19.3 | 17.6 | 20.8 | 19.7 |
| **8** | 1.5 | 2.3 | 17.2 | 2.3 | 2.8 | 3.2 | 2.7 | 0 | 14.7 | 14.2 | 15 | 15.6 | 16.1 | 16.1 | 17.7 | 15.5 | 18.3 | 17.9 |
| **9** | 15.4 | 16.9 | 2.6 | 16.9 | 17.4 | 17.6 | 17.2 | 14.7 | 0 | 0.48 | 1.3 | 1.3 | 1.5 | 1.5 | 3.1 | 0.92 | 4.2 | 3.3 |
| **10** | 15 | 16.3 | 3.0 | 16.3 | 16.9 | 17.1 | 16.7 | 14.2 | 0.48 | 0 | 1.2 | 1.8 | 1.9 | 1.9 | 3.5 | 1.4 | 4.7 | 3.7 |
| **11** | 15.8 | 17.2 | 2.5 | 17.2 | 17.7 | 18 | 17.5 | 15 | 1.3 | 1.2 | 0 | 0.61 | 1.5 | 1.5 | 3.2 | 1.5 | 3.5 | 3.4 |
| **12** | 16.5 | 17.8 | 1.9 | 17.8 | 18.3 | 18.6 | 18.1 | 15.6 | 1.3 | 1.8 | 0.61 | 0 | 0.92 | 0.92 | 2.6 | 1.2 | 3.5 | 2.8 |
| **13** | 16.9 | 18.3 | 1.5 | 18.3 | 18.7 | 18.7 | 18.2 | 16.1 | 1.5 | 1.9 | 1.5 | 0.92 | 0 | 0.41 | 1.9 | 0.58 | 3.4 | 2.0 |
| **14** | 16.9 | 18.3 | 1.5 | 18.3 | 18.7 | 18.7 | 18.2 | 16.1 | 1.5 | 1.9 | 1.5 | 0.92 | 0.41 | 0 | 2.0 | 1.0 | 3.3 | 2.2 |
| **15** | 18.5 | 19.7 | 0.97 | 19.7 | 19.9 | 19.8 | 19.3 | 17.7 | 3.1 | 3.5 | 3.2 | 2.6 | 1.9 | 2.0 | 0 | 2.1 | 2.6 | 0.33 |
| **16** | 16.4 | 17.7 | 1.9 | 17.7 | 18.2 | 18.1 | 17.6 | 15.5 | 0.92 | 1.4 | 1.5 | 1.2 | 0.58 | 1.0 | 2.1 | 0 | 3.8 | 2.3 |
| **17** | 19.1 | 20.5 | 1.9 | 20.5 | 21 | 21.3 | 20.8 | 18.3 | 4.2 | 4.7 | 3.5 | 3.5 | 3.4 | 3.3 | 2.6 | 3.8 | 0 | 2.6 |
| **18** | 18.7 | 20.1 | 1.0 | 20.1 | 20.2 | 20.1 | 19.7 | 17.9 | 3.3 | 3.7 | 3.4 | 2.8 | 2.0 | 2.2 | 0.33 | 2.3 | 2.6 | 0 |
