# Supplementary material for: Methodology of mixed load customized bus lines and adjustment based on time windows
Source: PLoS One. 2018 Jan 10;13(1):e0189763. doi: 10.1371/journal.pone.0189763 (PMC5761835; doi:10.1371/journal.pone.0189763)
Supplement: S11 Table — (DOCX) [file pone.0189763.s012.docx]

**S11 Table. Number of Passengers Getting on or off in the Time Window.**

| **Stop** | **1** | **2** | **3** | **4** | **5** | **6** | **7** | **8** | **9** | **10** | **11** | **12** | **13** | **14** | **15** | **16** | **17** | **18** |
| --- | --- | --- | --- | --- | --- | --- | --- | --- | --- | --- | --- | --- | --- | --- | --- | --- | --- | --- |
|  | 66 | 91 | 8 | 3 | 5 | 8 | 54 | 9 | 20 | 13 | 36 | 46 | 39 | 16 | 28 | 11 | 9 | 10 |
