# Supplementary material for: Methodology of mixed load customized bus lines and adjustment based on time windows
Source: PLoS One. 2018 Jan 10;13(1):e0189763. doi: 10.1371/journal.pone.0189763 (PMC5761835; doi:10.1371/journal.pone.0189763)
Supplement: S12 Table — (DOCX) [file pone.0189763.s013.docx]

**S12 Table. The Optimal Result of the Model.**

| **stop** | **Visiting order of stop** | **Cumulative operating distance (km)** | **Cumulative capacity (people)** |
| --- | --- | --- | --- |
| **1st bus** | Ocean Side→Run Yuan of Ocean Side→East of Ta Ying Street→South of Da Yu Zi Intersection→South of Guang Shun Street→East of Fu Tong Street→Guo Feng Beijing→Rong Ke Gan Lan City→West of Bei Xiao He→Hong Tai East Street→Bei Xiao He→East of Wang Jing Bei Road→Guangze Road | 32.72 | 49 |
| **2nd bus** | Ocean Side→Small Temple→West of Shuang Qiao→South of Da Yu Zi Intersection→East of Fu Tong Street→West of Bei Xiao He→Guangze Road | 23.44 | 49 |
| **3rd bus** | East of Chaoyang Mong Kok District→Hua Yu Yuan of Ocean Side→South of Guang Shun Street→West of Bei Xiao He→Guangze Road | 23.47 | 49 |
| **4th bus** | East of Chaoyang Mong Kok District→Ocean Side→Wang Ye Fen→Guo Feng Beijing→Hong Tai East Street→East of Wang Jing Bei Road | 23.78 | 49 |
| **5th bus** | Small Temple→South of Guang Shun Street→Guo Feng Beijing→Guangze Road | 21 | 40 |
